# (*E*)-2-[(2-Amino­phen­yl)imino­meth­yl]-4,6-di-*tert*-butyl­phenol

**DOI:** 10.1107/S1600536812038615

**Published:** 2012-09-15

**Authors:** Liqin Ding, Xingqiang Lü, Shunsheng Zhao, Yuqin Zhu

**Affiliations:** a School of Chemistry and Chemical Engineering, Xi’an Shiyou University, Xi’an 710065, Shaanxi, People’s Republic of China; b College of Chemical Engineering, Northwest University, Xi’an 710069, Shaanxi, People’s Republic of China; c College of Chemistry and Chemical Engineering, Xi’an University of Science and Technology, Xi’an 710054, Shaanxi, People’s Republic of China

## Abstract

In the title compound, C_21_H_28_N_2_O, the dihedral angle between the rings is 35.2 (2)°. A weak intra­molecular O—H⋯N hydrogen bond is observed between the O—H H atom and the imine N atom. In the crystal, mol­ecules are linked by additional inter­molecular N—H⋯O hydrogen bonding, resulting in a wave-like chain along the *b*-axis direction.

## Related literature

For related structures, see: Kochem *et al.* (2010 ▶); Belmonte *et al.* (2010 ▶); Liu *et al.* (2010 ▶). Details of the synthesis can be found in Muñoz-Hernández *et al.* (2000 ▶).
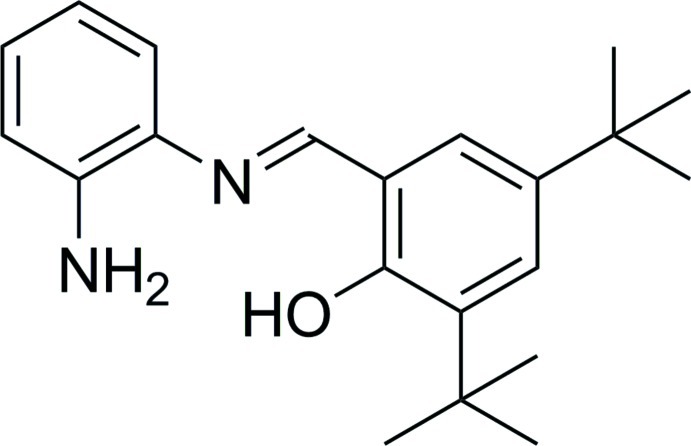


## Experimental

### 

#### Crystal data

C_21_H_28_N_2_O
*M* *_r_* = 324.45
Monoclinic, 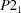

*a* = 10.898 (5) Å
*b* = 6.230 (3) Å
*c* = 15.095 (8) Å
β = 108.928 (6)°
*V* = 969.5 (8) Å^3^
*Z* = 2
Mo *K*α radiation
μ = 0.07 mm^−1^
*T* = 296 K
0.38 × 0.26 × 0.20 mm

#### Data collection

Bruker SMART 1K CCD area-detector diffractometer
Absorption correction: multi-scan (*SADABS*; Sheldrick, 2004 ▶) *T*_min_ = 0.979, *T*_max_ = 0.986
3718 measured reflections
1357 independent reflections
1224 reflections with *I* > 2σ(*I*)
*R*_int_ = 0.025
θ_max_ = 22.2°

#### Refinement

*R*[*F* ^2^ > 2σ(*F* ^2^)] = 0.044
*wR*(*F* ^2^) = 0.129
*S* = 1.10
1357 reflections
218 parameters
1 restraint
H-atom parameters constrained
Δρ_max_ = 0.22 e Å^−3^
Δρ_min_ = −0.15 e Å^−3^

### 

Data collection: *SMART* (Bruker, 2001 ▶); cell refinement: *SAINT* (Bruker, 2001 ▶); data reduction: *SAINT*; program(s) used to solve structure: *SHELXS97* (Sheldrick, 2008 ▶); program(s) used to refine structure: *SHELXL97* (Sheldrick, 2008 ▶); molecular graphics: *SHELXTL* (Sheldrick, 2008 ▶); software used to prepare material for publication: *SHELXTL* and local programs.

## Supplementary Material

Crystal structure: contains datablock(s) I, global. DOI: 10.1107/S1600536812038615/nc2289sup1.cif

Structure factors: contains datablock(s) I. DOI: 10.1107/S1600536812038615/nc2289Isup2.hkl

Supplementary material file. DOI: 10.1107/S1600536812038615/nc2289Isup3.cml

Additional supplementary materials:  crystallographic information; 3D view; checkCIF report

## Figures and Tables

**Table 1:** Hydrogen-bond geometry (Å, °)

*D*—H⋯*A*	*D*—H	H⋯*A*	*D*⋯*A*	*D*—H⋯*A*
O1—H1*A*⋯N1	0.82	1.87	2.608 (4)	149
N2—H2*A*⋯O1^i^	0.86	2.54	3.342 (4)	155

Symmetry code: (i) 
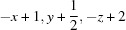
.

**Fig. 1.**
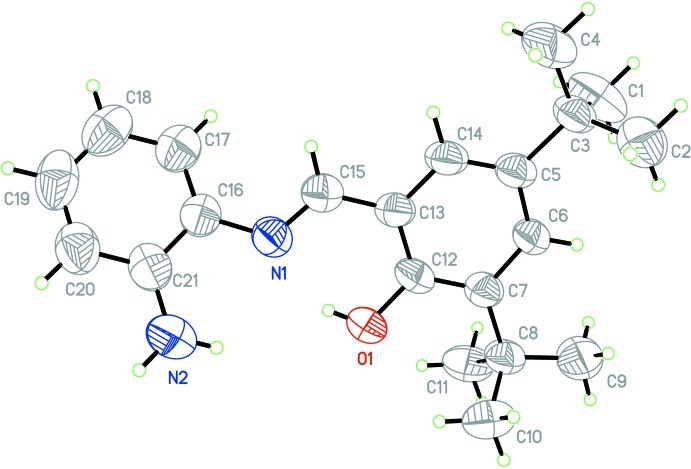
The molecular structure of the title compound with atom labels and 50% probability displacement ellipsoids for non-H atoms.

**Fig. 2.**
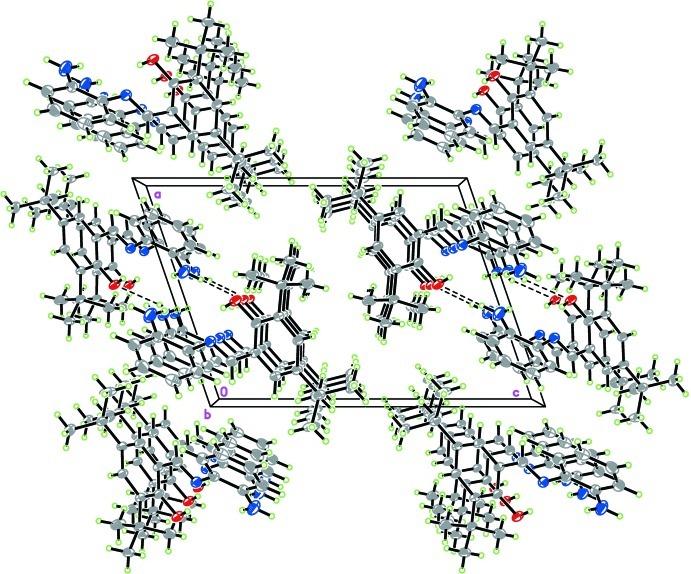
Crystal structure of the title compound with view along the b axis. Intermolecular hydrogen bonding is shown as dashed lines.

## supplementary crystallographic information

### Comment

The title compound is an important synthetic intermediate for design and synthesis of asymmetric Schiff base complexes showing excellent catalytic activity in various reactions. In the structure of the title compound the dihedral angle between the two phenyl rings amount to 35.2 (2) ° and all bond lengths are comparable to those observed in similar compounds (Kochem *et al.*, 2010; Belmonte *et al.*, 2010; Liu *et al.*, 2010) (Fig. 1). An intramolecular O—H···N hydrogen bond between the O-H H atom and the N atom N1 is observed (Table 1). In the crystal structure the molecules are linked into chains along the *b* axis by intermolecular N—H···O hydrogen bonding between the amino group and the hydroxy O atom which act as acceptor (Table 1 and Fig. 2).

### Experimental

The title compound was obtained according to the synthetic procedure of Muñoz-Hernández *et al.* (2000). 1,2-diaminobenzene (1.0 g, 9.2 mmol) was added to a solution of 3,5-Di-*tert*-butyl-2- hydroxybenzaldehyde (1.1 g, 4.6 mmol) in absolute ethanol (40 ml) and heated to reflux for 4 h, then concentrated to 20 ml by distillation. An orange solid precipitated from the reaction mixture and collected by filtration and dried open air. The orange solid was recrystalized from ethanol to give an orange crystal, which was collected by filtration and dried under vacuum, yield 71.0%. The single-crystal of the title compound suitble for X-ray diffraction was obtained by slow evaporation of an ethanol solution of the title compound.

### Refinement

Hydrogen atoms were positioned with idealized geometry (O-H H atoms allowed to rotate but no to tip) and refined using a riding model with N—H = 0.86 Å, C—H = 0.95–0.99 Å, O—H = 0.82 Å and with *U*_iso_(H) = 1.2 (1.5 for methyl groups) times *U*_eq_(C/N), *U*_iso_(H) = 1.5 *U*_eq_(O). Because no strong anomalous scattering atoms are present the absolute structure cannot be determined. Therefore, Friedel opposites were merged in the refinement.

### Crystal data

**Table:**

C_21_H_28_N_2_O	*F*(000) = 352
*M**_r_* = 324.45	*D*_x_ = 1.111 Mg m^−^^3^
Monoclinic, *P*2_1_	Mo *K*α radiation, λ = 0.71073 Å
Hall symbol: P 2yb	Cell parameters from 2451 reflections
*a* = 10.898 (5) Å	θ = 1.9–26.6°
*b* = 6.230 (3) Å	µ = 0.07 mm^−^^1^
*c* = 15.095 (8) Å	*T* = 296 K
β = 108.928 (6)°	Stick, orange
*V* = 969.5 (8) Å^3^	0.38 × 0.26 × 0.20 mm
*Z* = 2	

### Data collection

**Table:**

Bruker SMART 1K CCD area-detector diffractometer	1357 independent reflections
Radiation source: fine-focus sealed tube	1224 reflections with *I* > 2σ(*I*)
Graphite monochromator	*R*_int_ = 0.025
thin–slice ω scans	θ_max_ = 22.2°, θ_min_ = 2.0°
Absorption correction: multi-scan (*SADABS*; Sheldrick, 2004)	*h* = −11→11
*T*_min_ = 0.979, *T*_max_ = 0.986	*k* = −6→6
3718 measured reflections	*l* = −10→16

### Refinement

**Table:**

Refinement on *F*^2^	Secondary atom site location: difference Fourier map
Least-squares matrix: full	Hydrogen site location: inferred from neighbouring sites
*R*[*F*^2^ > 2σ(*F*^2^)] = 0.044	H-atom parameters constrained
*wR*(*F*^2^) = 0.129	*w* = 1/[σ^2^(*F*_o_^2^) + (0.0864*P*)^2^ + 0.0703*P*] where *P* = (*F*_o_^2^ + 2*F*_c_^2^)/3
*S* = 1.10	(Δ/σ)_max_ < 0.001
1357 reflections	Δρ_max_ = 0.22 e Å^−^^3^
218 parameters	Δρ_min_ = −0.15 e Å^−^^3^
1 restraint	Extinction correction: *SHELXL97* (Sheldrick, 2008), Fc^*^=kFc[1+0.001xFc^2^λ^3^/sin(2θ)]^-1/4^
Primary atom site location: structure-invariant direct methods	Extinction coefficient: 0.018 (7)

### Special details

**Table:**

Geometry. All e.s.d.'s (except the e.s.d. in the dihedral angle between two l.s. planes) are estimated using the full covariance matrix. The cell e.s.d.'s are taken into account individually in the estimation of e.s.d.'s in distances, angles and torsion angles; correlations between e.s.d.'s in cell parameters are only used when they are defined by crystal symmetry. An approximate (isotropic) treatment of cell e.s.d.'s is used for estimating e.s.d.'s involving l.s. planes.
Refinement. Refinement of *F*^2^ against ALL reflections. The weighted *R*-factor *wR* and goodness of fit *S* are based on *F*^2^, conventional *R*-factors *R* are based on *F*, with *F* set to zero for negative *F*^2^. The threshold expression of *F*^2^ > σ(*F*^2^) is used only for calculating *R*-factors(gt) *etc*. and is not relevant to the choice of reflections for refinement. *R*-factors based on *F*^2^ are statistically about twice as large as those based on *F*, and *R*- factors based on ALL data will be even larger.

### Fractional atomic coordinates and isotropic or equivalent isotropic displacement parameters (Å^2^)

**Table:**

	*x*	*y*	*z*	*U*_iso_*/*U*_eq_	
O1	0.5353 (2)	0.2087 (4)	0.80055 (16)	0.0705 (8)	
H1A	0.5635	0.3048	0.8389	0.106*	
N1	0.6999 (3)	0.4874 (5)	0.9023 (2)	0.0643 (8)	
C7	0.6019 (3)	−0.0180 (6)	0.6967 (2)	0.0588 (9)	
C6	0.7034 (3)	−0.0771 (7)	0.6648 (2)	0.0603 (9)	
H6A	0.6874	−0.1838	0.6193	0.072*	
C13	0.7507 (3)	0.2450 (6)	0.7952 (2)	0.0576 (9)	
C12	0.6285 (3)	0.1469 (6)	0.7643 (2)	0.0587 (9)	
C5	0.8277 (3)	0.0122 (7)	0.6961 (2)	0.0587 (9)	
C14	0.8484 (3)	0.1742 (7)	0.7605 (2)	0.0629 (10)	
H14A	0.9295	0.2392	0.7818	0.075*	
C3	0.9334 (3)	−0.0683 (7)	0.6571 (3)	0.0670 (10)	
C15	0.7804 (3)	0.4156 (6)	0.8635 (2)	0.0646 (10)	
H15A	0.8626	0.4771	0.8803	0.078*	
C8	0.4673 (3)	−0.1218 (7)	0.6579 (2)	0.0669 (11)	
C4	1.0650 (4)	0.0253 (13)	0.7108 (4)	0.126 (2)	
H4A	1.0608	0.1791	0.7071	0.189*	
H4B	1.1284	−0.0257	0.6841	0.189*	
H4C	1.0892	−0.0183	0.7752	0.189*	
C16	0.7344 (3)	0.6617 (6)	0.9657 (2)	0.0634 (10)	
C21	0.6777 (4)	0.6701 (8)	1.0365 (2)	0.0738 (11)	
C17	0.8160 (4)	0.8258 (7)	0.9580 (3)	0.0790 (12)	
H17A	0.8523	0.8223	0.9102	0.095*	
N2	0.5933 (4)	0.5117 (8)	1.0433 (3)	0.1113 (15)	
H2A	0.5580	0.5178	1.0866	0.134*	
H2B	0.5758	0.4066	1.0043	0.134*	
C20	0.7066 (4)	0.8395 (9)	1.0986 (3)	0.0887 (14)	
H20A	0.6707	0.8450	1.1466	0.106*	
C18	0.8438 (4)	0.9946 (8)	1.0206 (3)	0.0912 (13)	
H18A	0.8998	1.1033	1.0157	0.109*	
C2	0.9446 (5)	−0.3129 (9)	0.6651 (4)	0.1065 (16)	
H2C	0.8623	−0.3766	0.6316	0.160*	
H2D	0.9695	−0.3538	0.7298	0.160*	
H2E	1.0088	−0.3617	0.6388	0.160*	
C9	0.4638 (4)	−0.2935 (10)	0.5856 (4)	0.1117 (19)	
H9A	0.5266	−0.4026	0.6137	0.168*	
H9B	0.4838	−0.2300	0.5340	0.168*	
H9C	0.3789	−0.3564	0.5636	0.168*	
C19	0.7882 (4)	1.0003 (10)	1.0899 (3)	0.0961 (15)	
H19A	0.8059	1.1147	1.1317	0.115*	
C11	0.3664 (3)	0.0476 (9)	0.6095 (3)	0.0846 (14)	
H11A	0.3904	0.1140	0.5601	0.127*	
H11B	0.3624	0.1547	0.6543	0.127*	
H11C	0.2831	−0.0193	0.5838	0.127*	
C1	0.8970 (4)	−0.0129 (12)	0.5548 (3)	0.116 (2)	
H1C	0.8888	0.1400	0.5473	0.174*	
H1D	0.8158	−0.0796	0.5213	0.174*	
H1E	0.9631	−0.0636	0.5307	0.174*	
C10	0.4275 (4)	−0.2250 (8)	0.7362 (3)	0.0870 (13)	
H10A	0.4906	−0.3310	0.7675	0.130*	
H10B	0.3443	−0.2921	0.7101	0.130*	
H10C	0.4227	−0.1167	0.7802	0.130*	

### Atomic displacement parameters (Å^2^)

**Table:**

	*U*^11^	*U*^22^	*U*^33^	*U*^12^	*U*^13^	*U*^23^
O1	0.0581 (13)	0.0762 (17)	0.0891 (16)	−0.0028 (14)	0.0405 (13)	−0.0106 (15)
N1	0.0622 (17)	0.067 (2)	0.0671 (17)	0.0028 (16)	0.0262 (14)	−0.0019 (17)
C7	0.0492 (17)	0.062 (2)	0.0684 (19)	0.0013 (17)	0.0239 (15)	0.0010 (19)
C6	0.0470 (18)	0.068 (2)	0.070 (2)	−0.0007 (17)	0.0236 (15)	−0.010 (2)
C13	0.0491 (18)	0.062 (2)	0.0648 (19)	−0.0041 (16)	0.0224 (15)	0.0008 (18)
C12	0.0481 (18)	0.068 (2)	0.0668 (19)	0.0069 (17)	0.0280 (15)	0.004 (2)
C5	0.0445 (17)	0.065 (2)	0.069 (2)	0.0028 (18)	0.0222 (15)	0.003 (2)
C14	0.0468 (17)	0.071 (2)	0.074 (2)	−0.0040 (18)	0.0243 (16)	0.001 (2)
C3	0.050 (2)	0.075 (3)	0.081 (2)	0.0068 (19)	0.0296 (17)	0.001 (2)
C15	0.0577 (19)	0.068 (2)	0.069 (2)	−0.0031 (19)	0.0217 (17)	−0.001 (2)
C8	0.0463 (18)	0.081 (3)	0.076 (2)	−0.0073 (19)	0.0246 (16)	−0.008 (2)
C4	0.051 (2)	0.154 (5)	0.174 (5)	−0.001 (3)	0.039 (3)	−0.047 (5)
C16	0.0574 (19)	0.064 (2)	0.065 (2)	0.005 (2)	0.0137 (16)	−0.001 (2)
C21	0.065 (2)	0.088 (3)	0.065 (2)	0.009 (2)	0.0157 (18)	−0.005 (2)
C17	0.076 (2)	0.075 (3)	0.081 (3)	0.001 (2)	0.018 (2)	0.000 (2)
N2	0.110 (3)	0.137 (4)	0.110 (3)	−0.035 (3)	0.068 (2)	−0.031 (3)
C20	0.075 (3)	0.104 (4)	0.084 (3)	0.010 (3)	0.020 (2)	−0.018 (3)
C18	0.078 (2)	0.074 (3)	0.103 (3)	0.001 (2)	0.004 (2)	−0.002 (3)
C2	0.090 (3)	0.089 (3)	0.151 (4)	0.014 (3)	0.052 (3)	−0.001 (4)
C9	0.068 (2)	0.133 (5)	0.139 (4)	−0.033 (3)	0.039 (3)	−0.061 (4)
C19	0.088 (3)	0.098 (4)	0.083 (3)	0.017 (3)	0.001 (2)	−0.025 (3)
C11	0.0501 (19)	0.115 (4)	0.087 (3)	−0.003 (2)	0.0198 (18)	0.015 (3)
C1	0.096 (3)	0.162 (6)	0.111 (3)	0.032 (4)	0.063 (3)	0.026 (4)
C10	0.069 (2)	0.091 (3)	0.101 (3)	−0.014 (2)	0.027 (2)	0.013 (3)

### Geometric parameters (Å, º)

**Table:**

O1—C12	1.357 (4)	C21—N2	1.376 (6)
O1—H1A	0.8200	C21—C20	1.378 (6)
N1—C15	1.284 (4)	C17—C18	1.379 (6)
N1—C16	1.416 (5)	C17—H17A	0.9300
C7—C6	1.393 (4)	N2—H2A	0.8600
C7—C12	1.410 (5)	N2—H2B	0.8600
C7—C8	1.534 (5)	C20—C19	1.373 (7)
C6—C5	1.397 (4)	C20—H20A	0.9300
C6—H6A	0.9300	C18—C19	1.370 (6)
C13—C12	1.400 (5)	C18—H18A	0.9300
C13—C14	1.401 (5)	C2—H2C	0.9600
C13—C15	1.442 (5)	C2—H2D	0.9600
C5—C14	1.368 (5)	C2—H2E	0.9600
C5—C3	1.537 (5)	C9—H9A	0.9600
C14—H14A	0.9300	C9—H9B	0.9600
C3—C1	1.504 (6)	C9—H9C	0.9600
C3—C4	1.517 (6)	C19—H19A	0.9300
C3—C2	1.530 (7)	C11—H11A	0.9600
C15—H15A	0.9300	C11—H11B	0.9600
C8—C9	1.520 (6)	C11—H11C	0.9600
C8—C11	1.528 (6)	C1—H1C	0.9600
C8—C10	1.526 (6)	C1—H1D	0.9600
C4—H4A	0.9600	C1—H1E	0.9600
C4—H4B	0.9600	C10—H10A	0.9600
C4—H4C	0.9600	C10—H10B	0.9600
C16—C17	1.384 (6)	C10—H10C	0.9600
C16—C21	1.399 (5)		
			
C12—O1—H1A	109.5	C20—C21—C16	119.2 (4)
C15—N1—C16	120.3 (3)	C18—C17—C16	120.7 (4)
C6—C7—C12	116.3 (3)	C18—C17—H17A	119.6
C6—C7—C8	121.6 (3)	C16—C17—H17A	119.6
C12—C7—C8	122.0 (3)	C21—N2—H2A	120.0
C7—C6—C5	124.7 (3)	C21—N2—H2B	120.0
C7—C6—H6A	117.6	H2A—N2—H2B	120.0
C5—C6—H6A	117.6	C19—C20—C21	120.4 (4)
C12—C13—C14	119.6 (3)	C19—C20—H20A	119.8
C12—C13—C15	121.9 (3)	C21—C20—H20A	119.8
C14—C13—C15	118.5 (3)	C19—C18—C17	119.3 (5)
O1—C12—C13	119.8 (3)	C19—C18—H18A	120.4
O1—C12—C7	119.5 (3)	C17—C18—H18A	120.4
C13—C12—C7	120.6 (3)	C3—C2—H2C	109.5
C14—C5—C6	116.9 (3)	C3—C2—H2D	109.5
C14—C5—C3	122.8 (3)	H2C—C2—H2D	109.5
C6—C5—C3	120.3 (3)	C3—C2—H2E	109.5
C5—C14—C13	121.9 (3)	H2C—C2—H2E	109.5
C5—C14—H14A	119.1	H2D—C2—H2E	109.5
C13—C14—H14A	119.1	C8—C9—H9A	109.5
C1—C3—C4	110.4 (4)	C8—C9—H9B	109.5
C1—C3—C2	107.3 (5)	H9A—C9—H9B	109.5
C4—C3—C2	107.6 (4)	C8—C9—H9C	109.5
C1—C3—C5	109.7 (3)	H9A—C9—H9C	109.5
C4—C3—C5	111.5 (3)	H9B—C9—H9C	109.5
C2—C3—C5	110.2 (4)	C18—C19—C20	121.0 (5)
N1—C15—C13	123.7 (3)	C18—C19—H19A	119.5
N1—C15—H15A	118.2	C20—C19—H19A	119.5
C13—C15—H15A	118.2	C8—C11—H11A	109.5
C9—C8—C11	107.2 (3)	C8—C11—H11B	109.5
C9—C8—C10	108.2 (4)	H11A—C11—H11B	109.5
C11—C8—C10	108.6 (3)	C8—C11—H11C	109.5
C9—C8—C7	111.7 (3)	H11A—C11—H11C	109.5
C11—C8—C7	110.0 (4)	H11B—C11—H11C	109.5
C10—C8—C7	111.0 (3)	C3—C1—H1C	109.5
C3—C4—H4A	109.5	C3—C1—H1D	109.5
C3—C4—H4B	109.5	H1C—C1—H1D	109.5
H4A—C4—H4B	109.5	C3—C1—H1E	109.5
C3—C4—H4C	109.5	H1C—C1—H1E	109.5
H4A—C4—H4C	109.5	H1D—C1—H1E	109.5
H4B—C4—H4C	109.5	C8—C10—H10A	109.5
C17—C16—C21	119.4 (4)	C8—C10—H10B	109.5
C17—C16—N1	123.2 (3)	H10A—C10—H10B	109.5
C21—C16—N1	117.3 (4)	C8—C10—H10C	109.5
N2—C21—C20	120.6 (4)	H10A—C10—H10C	109.5
N2—C21—C16	120.2 (4)	H10B—C10—H10C	109.5

### Hydrogen-bond geometry (Å, º)

**Table:**

*D*—H···*A*	*D*—H	H···*A*	*D*···*A*	*D*—H···*A*
O1—H1*A*···N1	0.82	1.87	2.608 (4)	149
N2—H2*A*···O1^i^	0.86	2.54	3.342 (4)	155

Symmetry code: (i) −*x*+1, *y*+1/2, −*z*+2.

## References

[bb1] Belmonte, M. M., Wezenberg, S. J., Haak, R. M., Anselmo, D., Escudero-Adán, E. C., Benet-Buchholza, J. & Kleij, A. W. (2010). *Dalton Trans.* **39**, 4541–4550. 10.1039/b925560e 20372698

[bb2] Bruker (2001). *SMART* and *SAINT* Bruker AXS Inc., Madison, Wisconsin, USA.

[bb4] Kochem, A., Orio, M., Jarjayes, O., Neeseb, F. & Thomas, F. (2010). *Chem. Commun.* **46**, 6765–6767. 10.1039/c0cc01775b 20717604

[bb5] Liu, P., Feng, X. J. & He, R. (2010). *Tetrahedron*, **66**, 631–636.

[bb6] Muñoz-Hernández, M. A., Keizer, T. S., Parkin, S., Patrick, B. & Patrick, D. A. (2000). *Organometallics*, **19**, 4416–4421.

[bb7] Sheldrick, G. M. (2004). *SADABS* University of Göttingen, Germany.

[bb8] Sheldrick, G. M. (2008). *Acta Cryst.* A**64**, 112–122. 10.1107/S0108767307043930 18156677

